# Goal attainment in mobility after acute rehabilitation of mobility-restricting paralysis syndromes with regard to the ambulatory therapeutic level of participation NeuroMoves: A German national multicenter observational cohort study

**DOI:** 10.1186/s12883-021-02167-y

**Published:** 2021-04-07

**Authors:** Andreas Hug, Tamara Spingler, Cornelia Hensel, Stefan Fichtner, Tiziana Daniel, Laura Heutehaus, Michel Wensing, Rüdiger Rupp, Norbert Weidner

**Affiliations:** 1 Spinal Cord Injury Center, Heidelberg University Hospital, Schlierbacher Landstraße 200a, 69118 Heidelberg, Germany; 2 Department of General Practice and Health Services Research, Heidelberg University Hospital, Heidelberg, Germany

**Keywords:** Mobility, Participation, Stroke, Spinal cord injury, Physical therapy, Assistive devices, Ambulatory care, ICF

## Abstract

**Background:**

A central goal of rehabilitation in patients with paralysis syndromes after stroke or spinal cord injury (SCI) is to restore independent mobility as a pedestrian or wheelchair user. However, after acute rehabilitation, the mobility frequently deteriorates in the ambulatory setting, despite the delivery of rehabilitative interventions such as physical therapy or the prescription of assistive devices. The aim of the NeuroMoves study is to identify factors that are associated with changes of mobility in the ambulatory setting after acute inpatient rehabilitation, with a particular focus on participation according to the ICF (International Classification of Functioning, Disability and Health).

**Methods:**

The NeuroMoves study is intended as a national multicenter observational cohort study with 9 clinical sites in Germany. A total of 500 patients with mobility-restricting paralysis syndromes (i.e. stroke or SCI) are to be recruited during acute inpatient rehabilitation prior to discharge to the ambulatory setting. Patients will have 8 months of follow-up in the ambulatory setting. Three study visits at the clinical sites (baseline, midterm, and final) are planned at 4-months intervals. The baseline visit is scheduled at the end of the acute inpatient rehabilitation. During the visits, demographical data, neurological, functional, quality of life, and implementation measures will be assessed.

At baseline, each study participant receives an activity tracker (sensor for recording ambulatory mobility) along with a tablet computer for home use over the 8 months study duration. While mounted, the activity tracker records mobility data from which the daily distance covered by walking or wheelchair use can be calculated. Customized applications on the tablet computer remind the study participants to answer structured questionnaires about their health condition and treatment goals for physical therapy. Using the study participants’ tablet, therapists will be asked to answer structured questionnaires concerning treatment goals and therapeutic measures they have applied. The primary analysis concerns the association between mobility (daily distance covered) and the degree of participation-oriented rehab interventions. Further exploratory analyses are planned.

**Discussion:**

The findings could inform healthcare decision-making regarding ambulatory care in Germany focusing on mobility-promoting interventions for patients with mobility-restricting paralysis syndromes.

**Study registration:**

German Clinical Trials Register, DRKS-ID: DRKS00020487 (18.02.2020).

**Supplementary Information:**

The online version contains supplementary material available at 10.1186/s12883-021-02167-y.

## Background

Medical rehabilitation in Germany is largely regulated in a complementary way by two Social Codes (code of social law IX and V). Together with the social legislation for people with disabilities (code IX), the statutory health insurance (SHI) system (code V) aims to prevent, eliminate, reduce, and compensate for limited social participation or mitigate consequences thereof. The legislative wording underscores the importance of social participation in the context of rehabilitation. Within this framework of social legislation and the associated healthcare funds, the reimbursement of therapeutic interventions in medical rehabilitation depends on the type and severity of the health disorder as well as on the rehabilitation potential of the individual subject (e.g. physical therapy, occupational therapy, speech therapy, (neuro-)psychological, socio-medical, and nursing interventions) [1]. 73 million (88%) of the people in Germany are insured under SHI. By law, they are entitled to healthcare that is adequate, appropriate, and economical. The main principles for the provision of rehabilitation in the German social security system are “rehabilitation before retirement” and “rehabilitation before nursing”.

Concerning the setting of care, the German healthcare system is divided into an outpatient (ambulatory) and inpatient (hospital) sector. Ambulatory care is provided by general practitioners, specialists, dentists, and psychotherapists, as well as other healthcare professionals, such as physical therapists, orthopedic technicians, and occupational or speech therapists. Inpatient care is provided by around 1900 hospitals throughout Germany. Only a very small proportion of hospitals are also entitled to provide outpatient care (e.g. University outpatient clinics). Rehabilitation treatment after acute illnesses can be provided on either an outpatient or inpatient basis. For the acute phase of neurological disorders such as stroke or spinal cord injury (SCI), several weeks of inpatient neurorehabilitation in dedicated rehabilitation facilities are usually provided seamlessly after the acute hospital treatment. Due to several limitations concerning the consistency and continuity in the delivery of SHI health services, the subsequent between-sector transition (hospital to ambulatory care) has recently been reformed by legislation (“GKV-Versorgungsstärkungsgesetz”). Since 2017, hospitals have been obliged to implement discharge management programs. However, after hospital discharge, the quality and quantity of indicated neurorehabilitative interventions (e.g. physical therapy) after stroke and SCI is largely unknown. In the ambulatory sector particularly, dedicated health care managers/coordinators as well as models of multi-professional and interdisciplinary cooperation are missing [1].

Stakeholders of rehabilitation in Germany emphasize that health care interventions such as physical therapy should focus more on improvements in participation [1]. Therapeutic efforts in this regard only seem conceivable by comprehension and application of an integrative biopsychosocial model like the ICF (International Classification of Functioning, Disability and Health; https://www.who.int/classifications/drafticfpracticalmanual2.pdf?ua=1), which represents a conceptual framework for participation-oriented therapies. Within this biopsychosocial model, patients can be evaluated in a standardized and operationalized fashion. Moreover, rehabilitation procedures, as well as the prescription of mobility-promoting assistive devices, can be customized through the appreciation of individual and contextual factors [2, 3]. This can be achieved not only on the lower-dimensional level of body structure and function (e.g. reducing muscle tone), but also on the higher-dimensional levels of activities and participation (i.e. walking or mobility by other means, climbing stairs, extending the activity radius beyond the home, or the immediate surroundings [4]).

Frequently, chronic consequences of stroke and SCI lead to a permanent and severe restriction in mobility. Hence, one of the main treatment goals in acute and chronic rehabilitation after stroke and SCI represents the maintenance and improvement of mobility. Unfortunately, after the completion of acute rehabilitation in stroke survivors, the achieved mobility is deteriorating over the following 5 years after hospital discharge [5]. Moreover, disability-associated complications such as fractures after falls, or immobilization-related pressure injuries, which make it necessary to seek medical services, are common in the chronic course of stroke and SCI [6–8]. Several, yet only ill-defined implementation factors might contribute to this deterioration of mobility in the ambulatory sector:

1. Insufficient involvement of individual and contextual/environmental patient needs in defining treatment goals. The definition of treatment goals is mainly therapist-centered and on the level of body structure and function, while patients typically define their goals on the level of activities and participation (i.e. in a real-life situation). For example, physical therapists focus on measures aimed at reducing increased muscle tone (body function), while patients want to manage longer distances by foot or wheelchair to do daily errands (activities and participation). Prima facie, these different therapeutic views might lead to unequal expectations of therapists and patients and the impression that patient goals are only insufficiently pursued. In the last consequence, patient satisfaction and rehabilitative treatment success are negatively influenced [9, 10].
2. Physical therapy might be methodologically biased towards the level of body structure and function rather than the level of activities and participation. In Germany, physical therapy for patients with neurological disorders is mainly method−/technology-centered (e.g. special physical therapy for the treatment of central nervous system (CNS) disorders or SCI which uses the so-called neurophysiological techniques according to Bobath, Vojta, or Proprioceptive Neuromuscular Facilitation). These therapeutic interventions are reimbursed and operationalized according to the German therapy catalog (“Heilmittelkatalog”). Goal-oriented, therefore method−/technology-independent therapeutic interventions according to internationally consented neuroscientific principles are missing in the German “Heilmittelkatalog” [1, 11].
3. Coordination of rehabilitation-related health care interventions is only insufficiently implemented: Despite discharge management programs that are determined by legal regulation, the provision of mobility-promoting rehabilitative interventions like physical therapy and the prescription of assistive devices are only insufficiently coordinated, particularly in the ambulatory sector [1, 12].
4. Lack of indication-specific care pathways for the prescription of mobility-promoting assistive devices [1].
5. Systematic evaluations of goal attainment are not implemented in the current state of care [1].

To the best of our knowledge, there are no systematic analyses in large patient cohorts, neither nationally nor internationally, to investigate the implementation of ambulatory rehabilitation interventions. Moreover, association analyses between ambulatory rehabilitation interventions and pre-specified rehabilitation goals or objectively measured functional mobility outcomes are missing.

The NeuroMoves project aims for a systematic analysis of the current status of rehabilitation care at the transition between the hospital and ambulatory sectors with an emphasis on patient mobility. Moreover, the project intends to identify implementation deficiencies in the ambulatory delivery of rehabilitation services. The target population are people with mobility impairments following stroke or SCI over the first 8 months after acute rehabilitation.

## Objectives

The primary objectives are

1. The incongruence between treatment goals of health care providers (physician, physical therapy, occupational therapy, medical supply store) and patients with paralysis-related limitations of mobility (stroke, SCI) leads to a deterioration of their mobility.
2. Treatment goals of health care providers cannot be effectively achieved because

- assistive devices are not prescribed/administered according to the patients’ needs,
- physical therapy lacks sufficient participation-related patient goal orientation
- coordinated interdisciplinary cooperation of outpatient health care providers related to rehab interventions is missing.

## Methods

The outline of the methods section is oriented on the STROBE (Strengthening the Reporting of Observational Studies in Epidemiology) reporting guideline [13].

### Study design

We use an observational cohort design to follow-up stroke or SCI patients in the ambulatory sector for a fixed period of 8 months. Our main interest is on the organization and current practice of rehab interventions in the ambulatory sector in the real life setting after completion of acute rehabilitation. Therefore, patients are enrolled within 6 weeks after discharge.

### Recruitment

The target population recruitment takes place in two German regions, in the south and north (Rhein-Neckar region and Schleswig-Holstein/Hamburg states) between May 2020 and December 2021.

### Study centers

To adjust for clustering effects across Germany, four centers are in the north (Schleswig-Holstein and Hamburg) and five centers in the south (Rhein-Neckar area) of Germany. In addition to the coordinating center (Spinal Cord Injury Center of Heidelberg University Hospital), 8 other study centres are initiated and open for recruitment (Kliniken Schmieder, Heidelberg; Heinrich-Sommer-Klinik, Bad Wildbad; Sankt-Rochus-Klinik, Bad Schönborn; SRH Gesundheitszentrum Bad Wimpfen; Neurologisches Zentrum, Segeberger Kliniken GmbH, Bad Segeberg; August-Bier-Klinik, Bad Malente; Klinikum Bad Bramstedt GmbH, Bad Bramstedt; BG Klinikum Hamburg). Three centers are dedicated spinal cord injury centers and six centers are neurorehabilitation hospitals.

### Participants

Suitable study participants are to be identified, screened, and recruited in one of the 9 study centers at the end of acute rehabilitation.

#### Inclusion criteria

1. Patients with SCI or stroke at the end of initial inpatient treatment
2. Age between 18 and 85 years
3. At least minimal impairment of walking ability (score of ≤4 at item 8 in the mRMI): If the patient manages a flight of stairs independently (score of 5) without any aids or appliances, the patient cannot be enrolled
4. Wheelchair mobility (manual or electric wheelchair) achieved
5. Ability to follow study procedures (tablet usability, WLAN access guaranteed at least once a week)
6. Patients’ ability to consent
7. Written informed consent

#### Exclusion criteria

1. Serious psychiatric illness (e.g. schizophrenia, persistent suicidal tendencies)
2. Dependence on mechanical ventilation for respiration
3. Impairment of walking ability/mobility due to prior stroke
4. Lack of independent mobility prior to study entry (equivalent to mRS = 5)
5. Any physical or mental limitations that significantly restrict the adherence to the study procedures
6. Severe cognitive deficits

### Outcome measures

#### Primary outcomes

1. Objective measurement of mobility (walking distance and/or wheelchair distance) in everyday life of patients utilizing instrumented activity tracking in the ambulatory sector
2. Structured ICF-based assessment of rehabilitation care on mobility parameters in the ambulatory sector with particular focus on the ICF level of participation (i.e., in a real-life situation)
3. Evaluation of treatment goal agreement between patients and health care providers
4. Evaluation of treatment goal attainment in patients and health care providers, respectively

#### Secondary outcomes

1. Documentation of ambulatory mobility-related rehabilitation interventions (physical therapy, wheelchair features)
2. Assessment of the individual ambulatory rehabilitation care network of patients
3. Measuring the degree of coordination of health care interventions with a particular focus on ambulatory physical therapy

### Data collection

#### Data management system

**Fig. 1:**
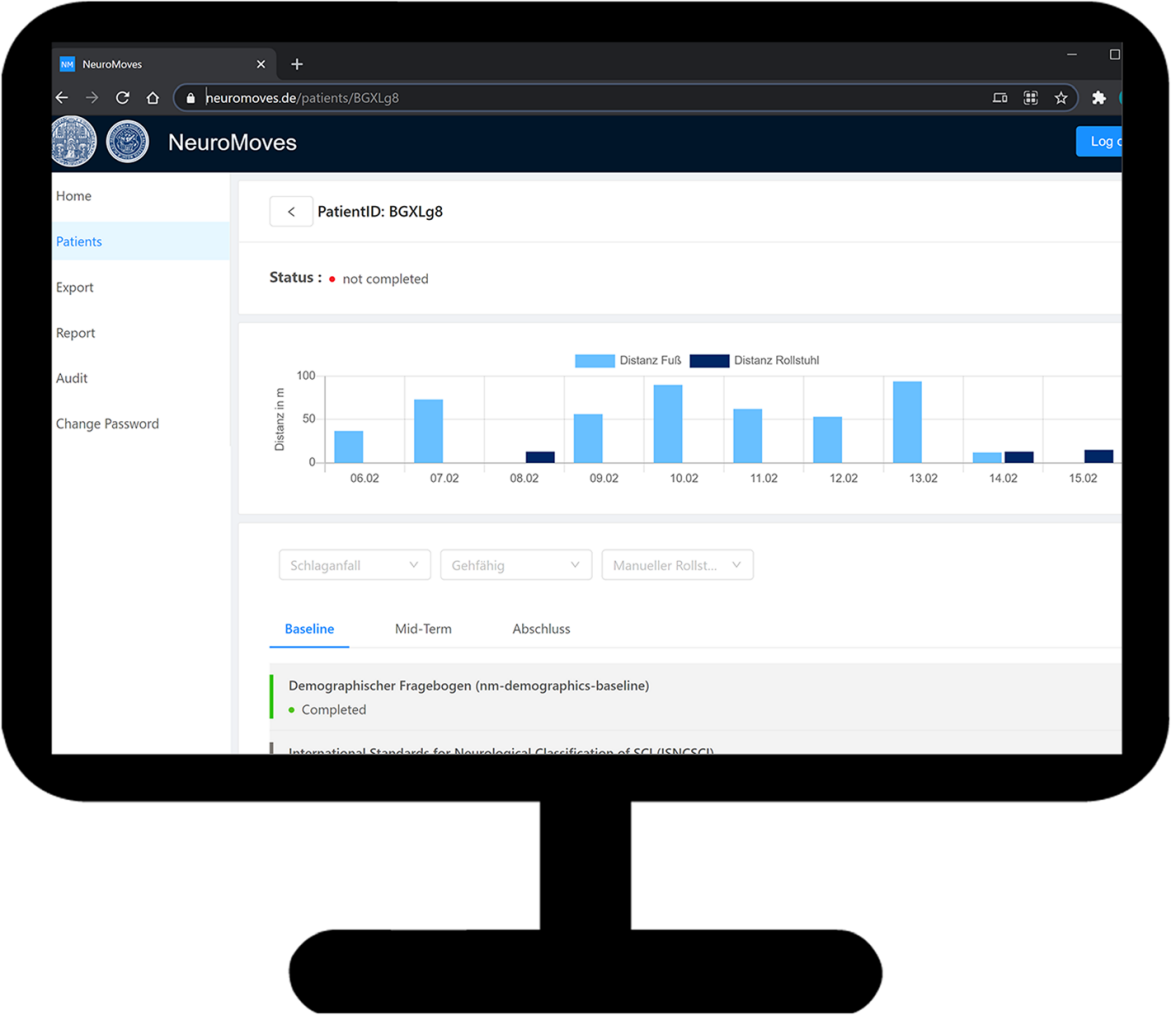
For illustration purposes the main page of the study management system which can be accessed via a web-client ist depicted. On the patients tab, the study subject can be selected using a pseudonymized patient ID. The investigators can follow-up on the mobility status over the last few days which is presented separately for distances covered by foot or wheelchair, respectively

The NeuroMoves project introduces a novel IT-supported patient management platform consisting of innovative monitoring tools based on instrumented activity tracking and web-based data collection options for patients and therapists. This platform is intended to ensure the most efficient and easy-to-handle data acquisition, technical and application-related interoperability, and data security for patients as well as health care service providers. This IT-platform forms the basis for a fast translation of the study results into a new standard of ambulatory care, where for example activity tracking might serve as an “early warning system” indicating a deterioration in mobility that might require medical attention. The results of the assessments collected at the visit time points are entered into the database by the investigators via a web client (Fig. 1). The IT-platform is fully compatible with the General Data Protection Regulation (GDPR).

#### Measures

**Fig. 2:**
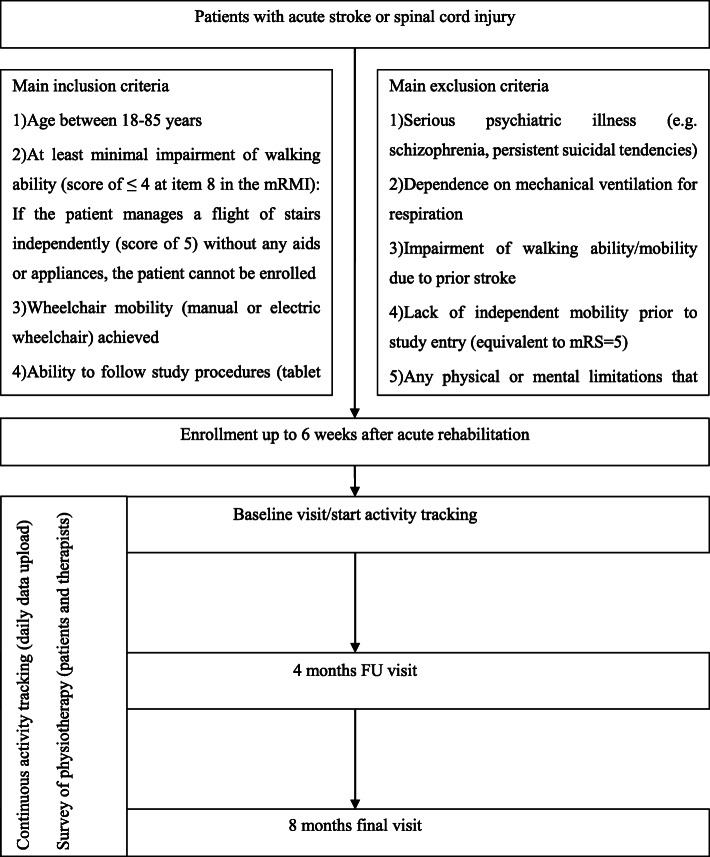
Study flowchart

After a potential candidate agrees to participate in the study, appropriate assessments (Table 1: NeuroMoves visit schedule; Fig. 2: study flowchart) are collected at three time points (baseline, midterm, final visit) within the 8-months observation period. The baseline visit takes place in the clinic at the end of inpatient care or within the first 6 weeks thereafter. For the midterm and final visits (and where required the baseline visit), study participants are asked to attend outpatient appointments at the respective study center.

**Table 1:** NeuroMoves visit schedule: within the 8-months observation period, the following assessments are collected at 3 visits: International Standards for Neurological Classification of Spinal Cord Injury (ISNCSCI), Spinal Cord Independence Measure (SCIM) III, National Institutes of Health Stroke Scale (NIHSS), modified Rankin Scale (mRS), Barthel Index (BI), functional independence measure (FIM), modified Rivermead Mobility Index (mRMI), 10-m walking test (10mWT), Timed up and go Test (TUG), Wheelchair Skills Test Questionnaire (WST-Q), Wheelchair Questionnaire, Depressions Anxiety and Stress Scale (DASS), World Health Organization Quality of Life Brief Version and disabilities module (WHOQOL-BREF-DIS), Questionnaire of individual care networks

Assessment	Baseline	Midterm 4 months ±2 weeks	Final 8 months ±2 weeks
**Demographics**	**X**	**X**	**X**
**Functional assessments/Questionnaires**			
**SCI**			
ISNCSCI: sensory and motor scores	**X**		**X**
SCIM	**X**		**X**
**Stroke**			
NIHSSS	**X**		**X**
mRS	**X**		**X**
**Stroke&SCI**			
BI	**X**	**X**	**X**
FIM	**X**	**X**	**X**
mRMI	**X**	**X**	**X**
10mWT	**X**	**X**	**X**
TUG	**X**	**X**	**X**
WST-Q	**X**	**X**	**X**
Wheelchair questionnaire	**X**	**X**	**X**
DASS	**X**	**X**	**X**
WHOQOL-BREF-DIS	**X**	**X**	**X**
Questionnaire and interview of individual care networks			**X**
**Training/Checkup**			
Activity tracker	**X**	**X**	**X**
Tablet-computer	**X**	**X**	**X**

### Instrumented activity tracking

**Fig. 3:**
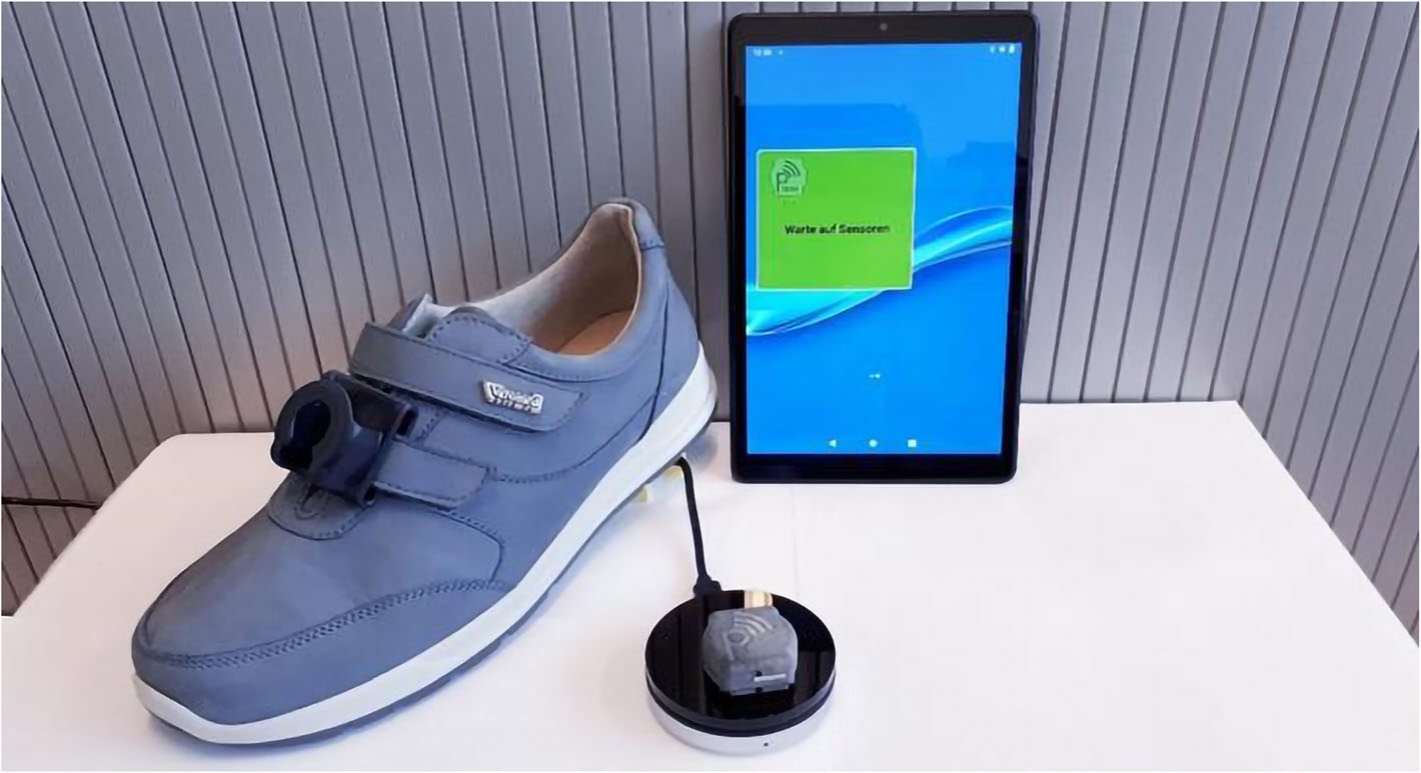
Technology for activity tracking: tablet-computer, sensor with wireless charger and sensor clip for attachment to shoe

During the baseline visit, study participants will receive a sensor and a tablet-computer for activity tracking (Fig. 3). While the tablet is needed only once a day to transmit the accumulated sensor data, the sensor must be permanently fixed to the shoe or the wheelchair to capture the activity of a whole day. The distance traveled per day is calculated by validated algorithms utilizing the raw data of an inertial motion sensor (IMU) integrating a triaxial accelerometer (acceleration) and a gyroscope (orientation/rotational movement).

Mobility data can be monitored after upload via the study management system by the responsible investigators. In case, the captured mobility data deviates massively from baseline values, or no data is uploaded over a substantial period, the responsible investigator will contact the study participant by email or phone to identify possible reasons.

Mobility patterns with respect to the proportion of safe/unsafe walkers or predominant wheelchair users in the ambulatory sector after stroke or SCI have not been systematically analyzed. In our expert opinion, three major mobility patterns can be expected among the study participants:

- Predominant and safe walkers (hereafter referred to as pedestrians) who do not need a wheelchair or use it only on very rare occasions. These are, for example, stroke patients with mild hemiparesis, who usually walk independently and do not use a wheelchair.
- Predominant wheelchair users (hereafter referred to as wheelchair users), who are not able to walk at all or only in a therapeutic setting. These are, for example, people with complete loss of function of the lower extremities after severe SCI or severe hemiparesis after stroke.
- Study participants who are capable of both, walking and using the wheelchair to achieve mobility (hereafter referred to as borderline walkers). These are people with e.g. incomplete SCI or moderate hemiparesis, who walk over short distances and use the wheelchair for longer distances).

To minimize measurement inaccuracies for the sensor-based algorithms for movement or step detection, the sensor of the activity tracker is attached as follows: For wheelchair users, a clip device is attached to the wheel of the wheelchair allowing for easy fixation and removal of the sensor. For pedestrians, the sensor is attached to the shoelaces or shoe strips on the less affected leg via a clip device. In the case of borderline walkers, two clip devices are used: One is attached to the shoe of the less affected leg and the other to the wheel of the wheelchair. Since only one sensor is available per study participant, the study participants are requested to put the sensor into the clip of the device in use (either shoe or wheelchair). Based on the motion profiles recorded by the sensor, the activity tracker automatically recognizes the type of mobility (pedestrian or wheelchair).

At the end of each day, study participants must recharge the sensor by removing it from the clip and placing it on a wireless charger. If the sensor and the tablet computer are within Bluetooth transmission range during the charging process, the sensor data is automatically uploaded to the tablet. A wireless internet connection is required to upload the activity data from the tablet to the server of the study management system. Usually, this upload is scheduled once a day, but can also be delayed up to a week. The study participants are instructed accordingly at the baseline visit. Every 2 weeks, study participants are contacted by phone to clarify technical questions and problems.

### Demographics

Identifying data, main and relevant secondary diagnoses are collected exclusively at the baseline visit. The coding of the main diagnosis (stroke or SCI) or secondary diagnosis is based on the ICD-10 version 2019.

### Assessment of neurological and functional impairment

To classify the neurological and functional impairment of the study participants, NIHSS [14] and mRS [15] will be applied at baseline and final visits for study participants with stroke. The International Standards for Neurological Classification of Spinal Cord Injury (ISNCSCI) [16] and the Spinal Cord Independence Measure (SCIM) III [17] are applied in study participants with SCI.

### Assessment of mobility

1. 10MWT, TUG
  The 10MWT (10-Meter Walk Test) is a mobility test that measures the time it takes a person to walk a distance of 10 m [18]. From this time, the self-selected walking speed is then determined. The TUG (Timed Up and Go test) is used to assess the risk of falling and the balance from sitting to standing and walking [19]. It uses the time that a person takes to rise from a chair, walk three meters, turn around, walk back to the chair, and sit down.
  The use of assistive devices is permitted in both tests and is documented. For patients, who are unable to walk, these tests cannot be performed.
  Both tests are validated for the assessment of mobility in both SCI [20] and stroke [21].
2. FIM
  The FIM (Functional Independence Measure) is a measuring instrument with 18 items for the assessment of motor, cognitive, and social functions, which are evaluated by the clinician. This measuring instrument was chosen with regard to the overall cohort because, unlike the SCIM (SCI only), it has been validated for use in both stroke and SCI. The FIM reflects the current functional status of a patient and not his/her functional capacity [22].
3. mRMI
  The mRMI (modified Rivermead Mobility Index) is a validated measuring instrument with 8 items for assessing mobility in stroke [23].
4. WST-Q
  The validated WST-Q (Wheelchair Skills Test-Questionnaire) objectively evaluates the safety and ability of patients to handle a manual or powered wheelchair [24–26]. It is only performed on patients who are dependent on a wheelchair.
5. Wheelchair questionnaire
  The questionnaire (not yet validated) created specifically for the NeuroMoves project serves to record the type of wheelchair and accessories used by study participants and is answered by the investigators. In order to record the satisfaction, the patient will answer questions regarding the importance of certain points (pain free sitting, independence and mobility) and his satisfaction with his wheelchair (supplementary file; questionnaire 1).
6. Barthel Index
  The Barthel Index (BI) is a method for evaluating the everyday abilities of a patient and is used to systematically assess independence and the need for care [27]. It has been validated for both patients with SCI and stroke.

### Assessment of depression and quality of life

1. DASS
  The DASS (Depression Anxiety Stress Scale) is a validated screening method and measures depression, anxiety, and stress levels in the past week [28].
2. WHOQOL-BREF-DIS
  The WHOQOL-BREF-DIS is an instrument for recording the subjective quality of life. It covers the areas of physical and mental well-being, social relations, and the environment. The DIS module deals with questions about physical limitations [29]. It has been validated in patients with SCI and stroke.

### Questionnaire and interview on the individual patient care network and coordination of care

During the final visit, the study participants will receive a questionnaire to indicate the individual patient care network and the degree of coordinated care. The questions relate to diseases other than stroke/SCI, the visited health care providers as inpatient and during the last 8 months in the home environment, circumstances of the physical therapy (e.g. process of finding the appropriate therapist), aspects regarding quality of therapy care, the cooperation between family practitioner and physical therapist and missed services (supplementary file; questionnaire 2).

Afterwards the participants will be invited to join an interview to explore the context and connections of factors which might influence the respective care in the ambulatory sector. The participants will be interviewed via telephone according to an interview guide. Data of the entire study sample is not manageable; therefore a purposive sampling strategy will be employed. The selection of participants is based on written consent, age, sex, stroke/SCI, study center (local distribution over Germany), mobility parameters and functional impairment. The data analysis follows the concept of qualitative content analysis [30].

### Patient-reported treatment goal attainment

**Fig. 4:**
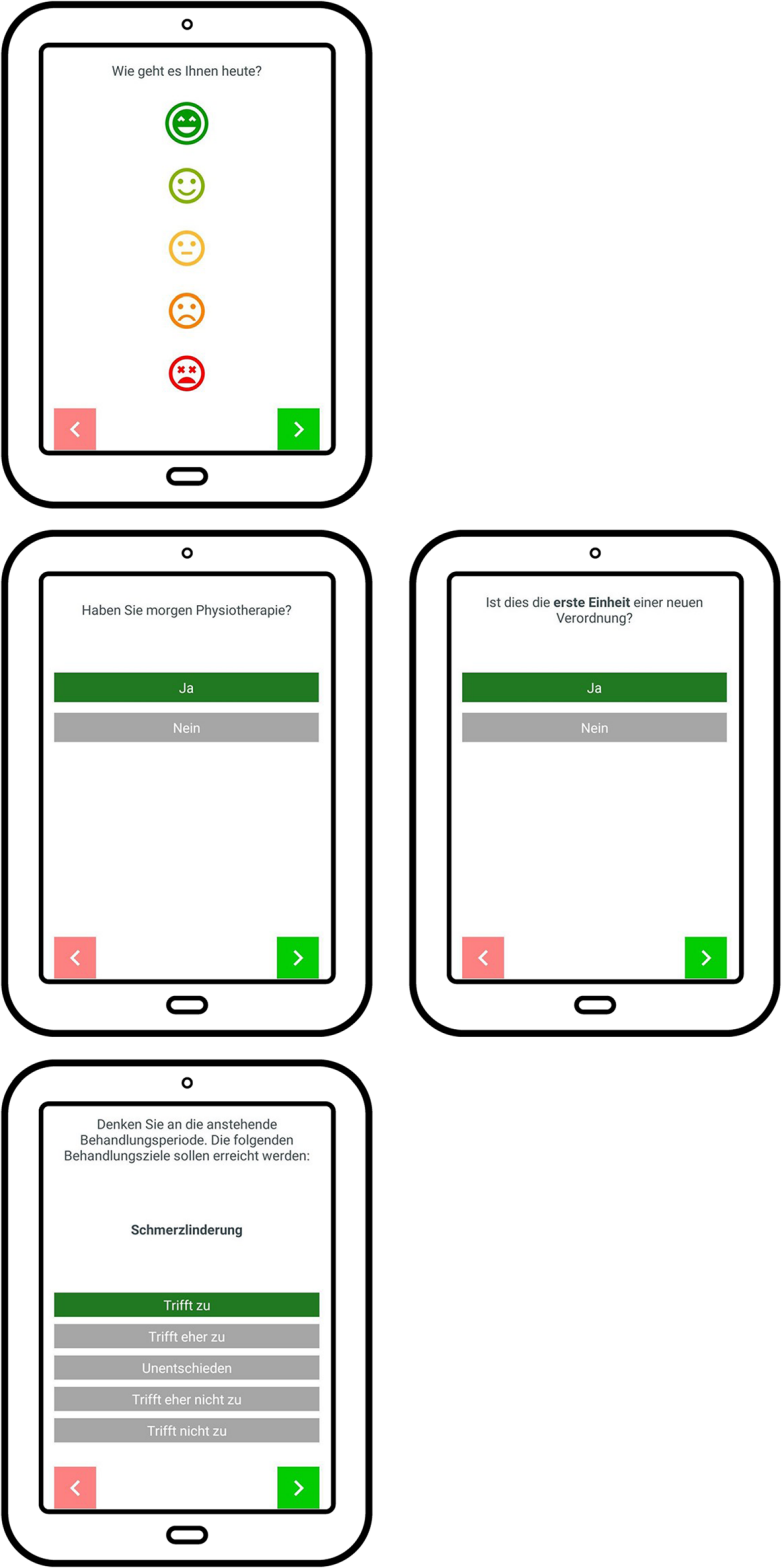
Screenshots of the patient App on the tablet computer. Upper panel: “evening query”. Mid panel (left): the study participant is asked whether an appointment for physical therapy is scheduled the following day. Mid panel (right): If it will be the first session of a new prescription cycle an 11-item (5-point ordinal scale for each item) questionnaire will pop up (lower panel) to select between 11 prespecified treatment goals (e.g. pain reduction)

To record the prescriptions of physical therapy, a daily interview is automatically generated by the tablet application (Fig. 4). This so-called “evening query” starts with the recurring question of “How are you today?”, which can be rated on a 5-point ordinal scale. Next, the study participant is asked whether an appointment for physical therapy is scheduled the following day. If it will be the first session of a new prescription cycle, an 11-item (5-point ordinal scale for each item) questionnaire will pop up to select between 11 prespecified treatment goals (joint mobility, pain reduction, spasticity in both legs, strength and endurance, transfer, standing and balance, walking, wheelchair mobility, 3 items about mobility). If it will be the last session of a prescription cycle, the participant will be asked to evaluate the same 11 items. Moreover, the participant will be asked to rate the overall satisfaction of goal attainment on a 5-point ordinal scale. Where applicable, the query algorithm reminds the study participant to bring along the tablet to the next physical therapy session to ask for corresponding ratings by the therapist (supplementary file; questionnaire 3).

### Therapist-reported treatment goal attainment and treatment measures

Similarly, the physical therapists are asked and evaluated about treatment goals for the upcoming prescription cycle on this 11-item questionnaire via the tablet of the participant (joint mobility, pain reduction, spasticity in both legs, strength and endurance, transfer, standing and balance, walking, wheelchair mobility, 3 items about mobility). Additionally, at the last treatment session, the therapist will be asked about the treatment setting (controlled environment (e.g. physical therapy practice), real-life simulation environment (e.g. stairs in physical therapy practice), or real-life situation (e.g. participants’ home, public transportation) and the applied treatment measures (e.g. joint mobilization, endurance training, strength training, sitting balance, standing balance, gait training, transfer−/wheelchair-training, coordination). Finally, the therapist will be asked to quantify the training intensity (dose) on a visual analogue scale (supplementary file; questionnaire 4).

### Statistical procedures

Primary outcome (1), which represents the basis for the sample size calculation, will be analyzed using multiple linear mixed regression methods for repeated measures. For these regression analyses, the primary outcome (1) is defined as the change in the daily distance covered between baseline and final visit after 8 months. To account for day-to-day fluctuations as well as for acclimatization effects during the transition between acute rehabilitation and settling into the home environment, the baseline mobility measurement is defined as the daily distance averaged over the second week after start of mobility recording, i.e. day 8–14 after baseline (up to 6 weeks after discharge from acute rehabilitation). Because we expect a significant number of participants in older age groups with difficulties to master the required technology, this approach allows for an extra week of getting accustomed to the activity tracker technology in both groups. For the final mobility measurement, we will use the daily distance averaged over the final week prior to the last study visit. The utilization of such statistical models allows for adjustments of confounding variables (e.g. daily mobility at baseline, number of treatment sessions, or severity of the neurological disorder). Moreover, linear mixed models are also quite robust and statistically more powerful with respect to missing data of repeated measurements (under the assumption of missing at random). Additionally, hitherto not further prespecified statistical analyses on the repeated mobility measurements are planned (e.g. individual continuous trajectories, bouts of mobility).

As the main predictor variable for the primary outcome (1) and basis for our sample size considerations, we will use the primary outcome (2) “degree of participation” of the physical therapy, which has been carried out during the 8 months study period. The answers of the therapist at the end of each prescription cycle to the itemized questionnaire on the patient tablet are the basis for an evaluation of the “degree of participation” of therapy, ranging from 0 to 100% of all treatment session.

Primary outcome (3) focuses on the agreement of treatment goals between patients and therapists. The itemized questionnaires between patients and therapists will be analyzed by intraclass-correlation methods. The resulting correlation coefficients indicate the degree of agreement concerning treatment goals between patients and therapists. Resulting agreement measures can serve for further exploratory analyses (e.g. subgrouping, use as confounding/explanatory variable in exploratory regression analyses, use as explanatory variable for quality of life measures, etc.).

Primary outcome (4) represents goal attainment analysis related to either patients or therapists, respectively. Subjectively highly ranked treatment goals are subject to requery at the end of each prescription cycle. The requery is performed via itemized questionnaires. Sum scores of items (item on 5-point ordinal scale) indicate the degree of goal attainment. Results serve for further exploratory analyses, e.g. as explanatory variable for functional outcomes, quality of life measures, etc.).

Secondary outcomes are evaluated with descriptive analysis.

### Sample size

The sample size calculation is based on the primary endpoint (1): the change of daily distance covered in meters between baseline and 8 months. The main predictor variable is the primary outcome (2) “degree of participation” on a scale from 0 to 100%. For study participants with SCI, the daily distance traveled is assumed to be 2000 m with a standard deviation of 2100 m on average, and for stroke survivors, the daily distance traveled is assumed to be 1586 m with a standard deviation of 1797 m on average [31, 32]. We assume that the study population will be composed of approximately 70% stroke survivors and 30% patients with SCI. We assume the same treatment effect of “the degree of participation” in both patient populations. For the overall sample size, the weighted mean is thus approximately 1700 m with a standard deviation of σY = 1900 m. From a standard deviation of the predictor of σX = 35.36 percentage points (based on the standard deviation of a non-informative Jeffrey’s Prior of the beta distribution, since there is no prior knowledge about the distribution of the predictor namely the “degree of participation”), it can be shown in a simple linear regression model with a case number of 500 patients that the regression coefficient βX for the main predictor differs from 0, assuming that under the alternative hypothesis the main predictor is at βX = 7.05 m of additionally traveled daily distance per percentage point of participation-oriented therapy (i.e. from 0 to 100% “degree of participation” would have an effect of approx. 700 m). We assume a dropout rate of 10%, a standard deviation of the primary outcome of 1900 m, a power of 80%, and a two-sided significance level of 5%. Using a linear mixed model and the addition of further positively correlated primary outcome measures is expected to increase power. This also applies to the additional inclusion of control variables as independent predictors in the statistical model, which we expect to explain an additional portion of the variance and thus lead to increased power. The number of cases was planned according to the method of Neter et al. [33] and the software PASS 14.0.

## Discussion

The study will evaluate factors that are associated with changes of mobility in the ambulatory setting over 8 months after discharge from acute rehabilitation in stroke and SCI patients. NeuroMoves is intended as a national multicenter observational cohort study with 9 clinical sites in Germany. Through the analysis of the current status of care at the transition between hospital and ambulatory care, the project intends to identify implementation insufficiencies in the ambulatory delivery of rehabilitation services with a particular focus on the therapeutic level of participation according to the ICF. To the best of our knowledge, there are no systematic analyses in large patient cohorts, neither nationally nor internationally, to investigate the implementation of ambulatory rehabilitation interventions.

The findings could inform healthcare decision-making regarding ambulatory care in Germany focusing on mobility-promoting interventions for patients with mobility-restricting paralysis syndromes.

## Supplementary Information

**Additional file 1.** Questionnaires developed for this study.

## Data Availability

Data sharing is not applicable to this article as no datasets were generated yet.

## Abbreviations

10mWT: 10-m walk test
ASIA: American Spinal Injury Association
BI: Barthel Index
CNS: Central nervous system
DASS: Depressions-Angst-Stress-Skalen
FIM: Functional Independence Measure
GB-A: Gemeinsamer Bundesausschuss
ICD-10: International Classification of Diseases - Version 10
ICF: International Classification for Functioning, Disability and Health
ICH-GCP: International Council for Harmonisation - good clinical practice
IMU: Inertial motion sensor
ISNCSCI: International Standards for Neurological Classification of Spinal Cord Injury
IT: Information Technology
mRMI: Modified Rivermead Mobility Index
mRS: Modified Rankin Score
NIHSS: National Institute of Health Stroke Scale
SCI: Spinal Cord Injury
SCIM: Spinal Cord Independence Measure
SGB: Sozialgesetzbuch
SOP: Standard Operating Procedure
TUG: Timed Up and Go Test
WHOQOL-BREF: World Health Organization Quality of Life abbreviated 26 item version
WHOQOL-DIS: World Health Organization Quality of Life Disability Module
WLAN: Wireless Local Area Network
WST-Q: Wheelchair Skills Test – Questionnaire
