# Supplementary material for: Goal attainment in mobility after acute rehabilitation of mobility-restricting paralysis syndromes with regard to the ambulatory therapeutic level of participation NeuroMoves: A German national multicenter observational cohort study
Source: BMC Neurol. 2021 Apr 7;21:149. doi: 10.1186/s12883-021-02167-y (PMC8025358; doi:10.1186/s12883-021-02167-y)
Supplement: Supplementary file 1 — Additional file 1. Questionnaires developed for this study. [file 12883_2021_2167_MOESM1_ESM.docx]

researchers' translation, no validated translation

# Questionnaire 1: Wheelchair questionnaire (implemented into App)

## Visit: Baseline

### Therapist

| Which wheelchair is the patient provided with? | ● Mechanical |
| --- | --- |
|  | ● E-wheelchair |
|  | ● Both |
| If the patient is provided with a mechanical wheelchair, does the patient use an auxiliary drive (e.g., wheel hub motor, inertia booster, traction device)? | Yes/No |
| What mechanical wheelchair does the patient use? | ● Active wheelchair |
|  | ● Lightweight wheelchair |
|  | ● Nursing wheelchair |
|  | ● Other |
| Does the patient use a one-hand drive? | Yes/No |
| Is the patient provided with a seat support? | Yes/No |
| Is the patient provided with an anti-decubitus cushion? | Yes/No |
| Has the patient been provided with the wheelchair that was prescribed? | ● Yes |
|  | ● No |
|  | ● Not supplied yet |
| Is the wheelchair custom-made | Yes/No |

### Patient

| Approximately how long do you sit in the wheelchair per day? | | | 0-24h | | | |
| --- | --- | --- | --- | --- | --- | --- |
| Is your sitting time limited by lack of assistance with transfers | | | Yes/No | | | |
| Have you had/do you have a decubitus ulcer in the ischial or coccygeal region? | | | Yes/No | | | |
| Did you perform wheelchair training? | | | Yes/No | | | |
| Do you experience pain when sitting? | | | Yes/No | | | |
| Do you propel the wheelchair mainly by yourself? | | | Mainly by yourself/mainly pushed | | | |
| Overall, are you satisfied with the wheelchair? | | | 1(very satisfied) - 5 (not at all satisfied). | | | |
| Goals:  As you look at the next 4 months, how important are the following to you in terms of your wheelchair care? | | | | | | |
|  | Very important | Rather important | | Partly/Partly | Rather not important | Not at all important/ already achieved/ unachievable |
| Sitting in a wheelchair without pain |  |  | |  |  |  |
| Independent wheelchair transfer |  |  | |  |  |  |
| independent  mobility in the  apartment/house |  |  | |  |  |  |
| independent mobility directly around the  apartment/house (e.g., garden, sidewalk in front of apartment/house) |  |  | |  |  |  |
| independent mobility beyond the apartment/house (e.g., to go shopping, to the doctor, to work) |  |  | |  |  |  |

## Visit midterm

### Therapist

Does the patient have a new wheelchair or have any changes been made to the care? If no, there are no further questions.

If yes:

| Which wheelchair is the patient provided with? | ● Mechanical |
| --- | --- |
|  | ● E-wheelchair |
|  | ● Both |
| If the patient is provided with a mechanical wheelchair, does the patient use an auxiliary drive (e.g., wheel hub motor, inertia booster, traction device)? | Yes/No |
| What mechanical wheelchair does the patient use? | ● Active wheelchair |
|  | ● Lightweight wheelchair |
|  | ● Nursing wheelchair |
|  | ● Other |
| Does the patient use a one-hand drive? | Yes/No |
| Is the patient provided with a seat support? | Yes/No |
| Is the patient provided with an anti-decubitus cushion? | Yes/No |
| Has the patient been provided with the wheelchair that was prescribed? | ● Yes |
|  | ● No |
|  | ● Not supplied yet |
| Is the wheelchair custom-made | Yes/No |
| Has the original wheelchair been adapted? | Yes/No  If Yes:  ● Does not fit  ● Deterioration of function  ● Improvement of function  ● Change in seating position  ● Pain  ● Other |
| Have the rental wheelchair been replaced by own wheelchair | Yes/No |

### Patient

| Approximately how long do you sit in the wheelchair per day? | | | 0-24h | | | |
| --- | --- | --- | --- | --- | --- | --- |
| Is your sitting time limited by lack of assistance with transfers | | | Yes/No | | | |
| Have you had/do you have a decubitus ulcer in the ischial or coccygeal region? | | | Yes/No | | | |
| Did you perform wheelchair training? | | | Yes/No | | | |
| Do you experience pain when sitting? | | | Yes/No | | | |
| Do you propel the wheelchair mainly by yourself? | | | Mainly by yourself/mainly pushed | | | |
| Overall, are you satisfied with the wheelchair? | | | 1(very satisfied) - 5 (not at all satisfied). | | | |
| Evaluation:  Looking at the last 4 months, how well were the goals you set for yourself achieved? | | | | | | |
|  | Very important | Rather important | | Partly/Partly | Rather not important | Not at all important/ already achieved/ unachievable |
| Sitting in a wheelchair without pain |  |  | |  |  |  |
| Independent wheelchair transfer |  |  | |  |  |  |
| independent mobility in the apartment/house |  |  | |  |  |  |
| independent mobility directly around the  apartment/house (e.g., garden, sidewalk in front of apartment/house) |  |  | |  |  |  |
| independent mobility beyond the apartment/house (e.g., to go shopping, to the doctor, to work) |  |  | |  |  |  |
| Goals:  As you look at the next 4 months, how important are the following to you in terms of your wheelchair care? | | | | | | |
|  | Very important | Rather important | | Partly/Partly | Rather not important | Not at all important/ already achieved/ unachievable |
| Sitting in a wheelchair without pain |  |  | |  |  |  |
| Independent wheelchair transfer |  |  | |  |  |  |
| independent mobility in the apartment/house |  |  | |  |  |  |
| independent mobility directly around the apartment/house (e.g., garden, sidewalk in front of apartment/house) |  |  | |  |  |  |
| independent mobility beyond the apartment/house (e.g., to go shopping, to the doctor, to work) |  |  | |  |  |  |

## Final visit

### Therapist

Does the patient have a new wheelchair or have any changes been made to the care? If no, there are no further questions.

If yes: repetition of midterm questionnaire

### Patient

| Approximately how long do you sit in the wheelchair per day? | | | 0-24h | | | |
| --- | --- | --- | --- | --- | --- | --- |
| Is your sitting time limited by lack of assistance with transfers | | | Yes/No | | | |
| Have you had/do you have a decubitus ulcer in the ischial or coccygeal region? | | | Yes/No | | | |
| Did you perform wheelchair training? | | | Yes/No | | | |
| Do you experience pain when sitting? | | | Yes/No | | | |
| Do you propel the wheelchair mainly by yourself? | | | Mainly by yourself/mainly pushed | | | |
| Overall, are you satisfied with the wheelchair? | | | 1(very satisfied) - 5 (not at all satisfied). | | | |
| Evaluation:  Looking at the last 4 months, how well were the goals you set for yourself achieved? | | | | | | |
|  | Very important | Rather important | | Partly/Partly | Rather not important | Not at all important/ already achieved/ unachievable |
| Sitting in a wheelchair without pain |  |  | |  |  |  |
| Independent wheelchair transfer |  |  | |  |  |  |
| independent mobility in the apartment/house |  |  | |  |  |  |
| independent mobility directly around the apartment/house (e.g., garden, sidewalk in front of  apartment/house) |  |  | |  |  |  |
| independent mobility beyond the apartment/house (e.g., to go shopping, to the doctor, to work) |  |  | |  |  |  |

# Questionnaire 2: (paper form)

researchers' translation, no validated translation

## Part 1: Questions about yourself and medical history

| Your year of birth? |  |
| --- | --- |
| Your gender? | Female\|Male\|Divers |
| Are you a pedestrian, wheelchair user or both? | Pedestrian\|Wheelchair user\|Pedestrian and wheelchair user |
| What is the size of the town in which you reside? | Large city: > 100,000 inhabitants\|Medium-sized city: 20,000 to 100,000 inhabitants\|Small town: 5,000 to 20,000 inhabitants\|Rural town/urban community: 2,000 to 5,000 inhabitants\|Rural community: < 2,000 inhabitants |
| Which payer covers the costs for your medical care? | Statutory health insurance\|Private health insurance\| Employer's liability insurance\|Liability insurance\|Social welfare\|Other (please specify): |
| What chronic conditions did you have before your SCI? Please tick all applicable se options. | I did not have a chronic disease\|Joint disease (e.g. osteoarthritis, rheumatism)\|Herniated disc\|Bone disease (e.g. osteoporosis)\|Chronic back pain\|Chronic kidney disease\|Chronic lung disease (e.g. asthma, COPD)\|Chronic thyroid disease\|Chronic gastrointestinal disease\|Allergy/chronic skin disease\|Diabetes mellitus\|Depression or anxiety disorder\|Addictive disorder (e.g., alcohol dependence, nicotine)\|Cardiovascular disease (e.g. heart failure, high blood pressure)\|Neurological disease (e.g. Parkinson's disease, stroke)\|Cancer\|Other (please specify):… |
| What problems or secondary conditions due to your SCI do you suffer from? Please tick all applicable options. | I have no problems or secondary disease\|Joint pain, pain in everyday life\|Contractures (reduced joint mobility)\|Muscular spasticity (twitching, spasms, uncontrolled movements)\|Pressure sores (rashes, redness, wounds)\|Urinary tract infections (e.g., bladder and kidney infections)\|Chronic kidney disease (e.g., renal insufficiency)\|Urinary bladder dysfunction (e.g., incontinence, urinary retention)\|Bowel dysfunction (e.g., diarrhea, incontinence, constipation)\|Fatigue syndrome, chronic fatigue\|Joint disease (e.g., osteoarthritis, rheumatism)\|Chronic back pain\|Pulmonary disease, respiratory problems (e.g., pneumonia, shortness of breath)\|Gastrointestinal problems (e.g., gastritis)\|Depression or anxiety disorder\|Sleep disorders (e.g. problems with falling asleep or sleeping through the night)\|Addiction (e.g. alcohol dependence, nicotine)\|Circulatory disorders (swelling of the feet, legs or arms, thrombosis, embolism)\|Cardiovascular diseases (e.g. heart failure, high blood pressure)\|Vegetative regulation disorders (sudden increase in blood pressure, sweating, goose bumps, headaches, dizziness when changing position)\|Other (please specify): |
| Do you participate in a family doctor-centered care (HZV; Hausarzt-zentrierte Versorgung) program or similar program? | yes\|no\|don't know\|This refers to a primary care physician program in which the primary care physician coordinates health care, makes referrals to specialists, and collects all medical reports, among other things. |

## Part 2: Your individual healthcare network

| Which doctor handles most of your health care or is your prescribing doctor? | Family Physician\|Neurologist\|Other (please specify): | | | | | |
| --- | --- | --- | --- | --- | --- | --- |
|  | How many contacts have there been since discharge from rehab? | Have the contents of your healtcare been coordinated with your prescribing physician? | How important is this professional group as a contact for your SCI? | | | |
|  |  |  | Very important | important | Less important | Unclear/n.a. |
| General practitioner during consultation hours | None\|1 to 2\|More than 2 | Yes\|Partly\|No | o | o | o | o |
| General practitioner by telephone | None\|1 to 2\|More than 2 | Yes\|Partly\|No | o | o | o | o |
| Physician's assistant/clinical assistant at general practitioner | None\|1 to 2\|More than 2 | Yes\|Partly\|No | o | o | o | o |
| Healthcare assistant (VERAH) at general practitioner | None\|1 to 2\|More than 2 | Yes\|Partly\|No | o | o | o | o |
| Consultant neurologist during consultation hours | None\|1 to 2\|More than 2 | Yes\|Partly\|No | o | o | o | o |
| Consultant neurologist by telephone | None\|1 to 2\|More than 2 | Yes\|Partly\|No | o | o | o | o |
| Physician's assistant/clinical assistant at neurologist | None\|1 to 2\|More than 2 | Yes\|Partly\|No | o | o | o | o |
| Consultant orthopaedist during consultation hours | None\|1 to 2\|More than 2 | Yes\|Partly\|No | o | o | o | o |
| Consultant orthopaedist by telephone | None\|1 to 2\|More than 2 | Yes\|Partly\|No | o | o | o | o |
| Physician's assistant/clinical assistant at orthopaedist | None\|1 to 2\|More than 2 | Yes\|Partly\|No | o | o | o | o |
| Consultant internist / cardiologist during consultation hours | None\|1 to 2\|More than 2 | Yes\|Partly\|No | o | o | o | o |
| Consultant internist / cardiologist by telephone | None\|1 to 2\|More than 2 | Yes\|Partly\|No | o | o | o | o |
| Physician's assistant/clinical assistant at internist / cardiologist | None\|1 to 2\|More than 2 | Yes\|Partly\|No | o | o | o | o |
| Consultant (neuro-) urologist during consultation hours | None\|1 to 2\|More than 2 | Yes\|Partly\|No | o | o | o | o |
| Consultant (neuro-) urologist by telephone | None\|1 to 2\|More than 2 | Yes\|Partly\|No | o | o | o | o |
| Physician's assistant/clinical assistant at consultant (neuro-)urologist | None\|1 to 2\|More than 2 | Yes\|Partly\|No | o | o | o | o |
| Consultant ENT spacialist during consultation hours | None\|1 to 2\|More than 2 | Yes\|Partly\|No | o | o | o | o |
| Consultant ENT specialist by telephone | None\|1 to 2\|More than 2 | Yes\|Partly\|No | o | o | o | o |
| Physician's assistant/clinical assistant at consultant ENT spacialist | None\|1 to 2\|More than 2 | Yes\|Partly\|No | o | o | o | o |
| Lifelong follow-up (control examinations) at the (neuro-)urological outpatient clinic for SCI | None\|1 to 2\|More than 2 | Yes\|Partly\|No | o | o | o | o |
| Lifelong follow-up (check-ups) in a (non-neuro- urological) outpatient clinic for SCI | None\|1 to 2\|More than 2 | Yes\|Partly\|No | o | o | o | o |
| Lifelong follow-up (check-ups) in an out-patients department of specialist clinic | None\|1 to 2\|More than 2 | Yes\|Partly\|No | o | o | o | o |
| Psychologist or psychotherapist during consultation hours | None\|1 to 2\|More than 2 | Yes\|Partly\|No | o | o | o | o |
| Psychologist or psychotherapist by telephone | None\|1 to 2\|More than 2 | Yes\|Partly\|No | o | o | o | o |
| Assistant at psychotherapist's office | None\|1 to 2\|More than 2 | Yes\|Partly\|No | o | o | o | o |
| Consultant for physical and rehabilitative medicine during consultation hours | None\|1 to 2\|More than 2 | Yes\|Partly\|No | o | o | o | o |
| Consultant for physical and rehabilitative medicine by telephone | None\|1 to 2\|More than 2 | Yes\|Partly\|No | o | o | o | o |
| Physician's assistant/clinical assistant at consultant for physical and rehabilitative medicine | None\|1 to 2\|More than 2 | Yes\|Partly\|No | o | o | o | o |
| Physiotherapist in the physiotherapy practice | None\|1 to 2\|More than 2 | Yes\|Partly\|No | o | o | o | o |
| Occupational therapist in the therapy practice | None\|1 to 2\|More than 2 | Yes\|Partly\|No | o | o | o | o |
| Speech therapist in the therapy practice | None\|1 to 2\|More than 2 | Yes\|Partly\|No | o | o | o | o |
| Employees in the medical supply store | None\|1 to 2\|More than 2 | Yes\|Partly\|No | o | o | o | o |
| Rehab sport group(s)/IRENA program | None\|1 to 2\|More than 2 | Yes\|Partly\|No | o | o | o | o |
| Sports for the disabled in a sports club (e.g. wheelchair club, archery) | None\|1 to 2\|More than 2 | Yes\|Partly\|No | o | o | o | o |
| Leisure activities (e.g. afternoon meeting, singing club) | None\|1 to 2\|More than 2 | Yes\|Partly\|No | o | o | o | o |
| Pharmacist/Pharmacist | None\|1 to 2\|More than 2 | Yes\|Partly\|No | o | o | o | o |
| Ambulatory nursing service | None\|1 to 2\|More than 2 | Yes\|Partly\|No | o | o | o | o |
| Alternative therapies (e.g. alternative practitioner, acupuncture, osteopathy) | None\|1 to 2\|More than 2 | Yes\|Partly\|No | o | o | o | o |
| Local nursing care counseling center | None\|1 to 2\|More than 2 | Yes\|Partly\|No | o | o | o | o |
| Consultant/service employee of your health care cost payer (health insurance fund, employers' liability insurance association) (also by telephone) | None\|1 to 2\|More than 2 | Yes\|Partly\|No | o | o | o | o |
| Consultant/service employee of your pension insurance (also by phone) | None\|1 to 2\|More than 2 | Yes\|Partly\|No | o | o | o | o |
| Consultant/service employee of the medical service of the statutory health insurance system, MDK (also by telephone) | None\|1 to 2\|More than 2 | Yes\|Partly\|No | o | o | o | o |
| Interest group/network association/foundation or similar | None\|1 to 2\|More than 2 | Yes\|Partly\|No | o | o | o | o |
| Neighborhood assistance/domestic help/"meals on wheels” | None\|1 to 2\|More than 2 | Yes\|Partly\|No | o | o | o | o |
| Relatives/family/ trained person as assistant or caregiver | None\|1 to 2\|More than 2 | Yes\|Partly\|No | o | o | o | o |
| Municipal and community facilities (e.g. application for a car parking permit) | None\|1 to 2\|More than 2 | Yes\|Partly\|No | o | o | o | o |
| Other: (please specify) | None\|1 to 2\|More than 2 | Yes\|Partly\|No | o | o | o | o |

| The following is about your time (until about eight months ago) in acute inpatient treatment and inpatient rehabilitation due to your SCI: Which of the following health care providers did you have contact with? Which was particularly important to you for your ambulatory care? | | | | | |
| --- | --- | --- | --- | --- | --- |
| This includes any form of contact related to the provision of medical services and medical advice (e.g. examinations, treatments, ordering/collection of prescriptions, requests of reports). | | | | | |
| Ambulatory care includes, among other things, the provision of medical aids, assistive devices, physiotherapy, occupational therapy, medication, and care by general practitioners or specialists. | | | | | |
| ACUTE CARE CLINIC (HOSPITAL) | | | | | |
|  | Were there contacts in inpatient care? | How important was this professional group for your ambulatory care? | | | |
|  |  | Very important | Important | Less important | Unclear/n.a. |
| Ward physicians in the acute care clinic | Yes\|No | o | o | o | o |
| Neuro-urologist in acute care hospital | Yes\|No | o | o | o | o |
| Nursing in the acute care clinic | Yes\|No | o | o | o | o |
| Physiotherapists in the acute care clinic | Yes\|No | o | o | o | o |
| Occupational therapists in the acute care clinic | Yes\|No | o | o | o | o |
| Speech therapists in the acute care clinic | Yes\|No | o | o | o | o |
| Social service in the acute clinic | Yes\|No | o | o | o | o |
| Psychotherapist, (neuro-) psychologist or pastoral care in the acute care clinic. | Yes\|No | o | o | o | o |
| Dietician in acute care clinic | Yes\|No | o | o | o | o |
| Other at acute care clinic (please specify): | Yes\|No | o | o | o | o |
|  |  |  |  |  |  |
| REHABILITATION CLINIC (or continued rehabilitation in acute hospital) | | | | | |
|  |  |  |  |  |  |
|  | Were there contacts in inpatient care? | How important was this professional group for your ambulatory care? | | | |
|  |  | Very important | Important | Less important | Unclear/n.a. |
| Physicians in the rehabilitation clinic | Yes\|No | o | o | o | o |
| Neuro-urologist in rehabilitation clinic | Yes\|No | o | o | o | o |
| Nursing in the rehabilitation clinic | Yes\|No | o | o | o | o |
| Physiotherapists in the rehabilitation clinic | Yes\|No | o | o | o | o |
| Occupational therapists in the rehabilitation clinic | Yes\|No | o | o | o | o |
| Speech therapists in the rehabilitation clinic | Yes\|No | o | o | o | o |
| Social service in the rehabilitation clinic | Yes\|No | o | o | o | o |
| Psychotherapist, (neuro-) psychologist or pastoral care in the rehabilitation clinic | Yes\|No | o | o | o | o |
| Dietician in the rehabilitation clinic | Yes\|No | o | o | o | o |
| Other at rehab clinic (please specify): | Yes\|No | o | o | o | o |

## Part 3: Your ambulatory physiotherapy

We are interested in your experiences and impressions regarding your ambulatory physiotherapy treatments since discharge from the rehabilitation clinic. For each of the following statements, please mark the answer that best corresponds to your opinion.

| The following statements relate to your experience with physiotherapy practices. Your current physiotherapy practice is the one where you receive the most treatment for your SCI. Even if you are not currently receiving physiotherapy, please answer the questions related to your most recent physiotherapy practice. | |
| --- | --- |
| In general, how often per week or per month do you have physiotherapy? | Currently no physiotherapy\|Per week: x times\|OR per month x times |
| How often have you changed your physiotherapist/physiotherapy practice by yourself? | No change (please skip the next question)\|x times |
| For what reasons did you change the physiotherapist/physiotherapy practice by yourself? Please check all that apply. If you have not changed the physiotherapist or physiotherapy practice on your own, please skip this question. | Accessibility of the practice\|Therapy offered or therapy content\|Practice organization (e.g., appointment scheduling, frequent therapist changes, equipment)\|Lack of sympathy with the therapist\|Lack of expertise of the physiotherapist\|Monotone/ incomplete treatments\| Did not meet my expectations\|The treatments were not according to my wishes\| Other (please specify): |
| How did you or your family members found out your current physiotherapy practice? Please check all that apply. | Recommendation from other patients\|Recommendation from other physiotherapists\|Through experience of my (family) doctor\|Through experience of the rehabilitation clinic\|Through own previous experience, from relatives, friends or acquaintances\|By telephone or personal inquiry in the practice\| Through the practice's website\|Through information from other websites, from interest groups, professional associations, clubs or similar.\|Other (please spedify): |
| What criteria did you use to select your physiotherapy practice? Please check all that apply. | (Only one) practice close to home\|(Only one) barrier-free practice (handicapped parking, wheelchair ramps, door opening possible, toilet access)\|Practice organization (e.g. appointment allocation, assignment to the desired therapist)\|Offers specialized therapy\|practice equipment\|Professional experience of the physiotherapist\|Professional knowledge of the physiotherapist\|Sympathy with the therapist(s)\|Content of treatment\|Other (please specify): |
| How do you mainly reach your physiotherapy practice? | Physiotherapist comes to my home (home visits)\|By foot/by bike/by wheelchair\|By car/motorized scooter\|By public transport (with\|without companion) |
| How long does it usually take to get to your physiotherapy practice? Or: How long does it take your physiotherapist to get to your home? | Journey time: minutes\|One way distance: kilometers |

| The following statements relate to your physiotherapy prescriptions and treatment content. Which treatment methods have been prescribed? What physiotherapy content have you received so far? And how useful do you consider the prescriptions and contents of the treatments? The prescriptions and treatment contents refer exclusively to the time after your inpatient rehab. Even if you are not currently receiving physiotherapy, please answer the questions in relation to the physiotherapy treatments you have received. | | | | |
| --- | --- | --- | --- | --- |
| What kind of physiotherapy has been prescribed? | How useful do you think the prescription is for you? | | | |
|  | Very useful | Useful | Less useful | Unclear n.a. |
| I do not know |  | | | |
| Bobath | o | o | o | o |
| Vojta | o | o | o | o |
| PNF | o | o | o | o |
| KG (normal physiotherapy) | o | o | o | o |
| KG-G (device-supported physiotherapy) | o | o | o | o |
| Massage | o | o | o | o |
| Heat/cold/  electrotherapy | o | o | o | o |
| Manual lymphatic drainage | o | o | o | o |
| Other (please specify): | o | o | o | o |
| What content of physiotherapy have you received so far? | How useful do you consider the contents of physiotherapy to be for you? | | | |
| Passive movement of individual joints, improvement of joint mobility | o | o | o | o |
| Measures for pain relief | o | o | o | o |
| Activation / stimulation of muscles and nerves | o | o | o | o |
| Activation / stimulation of movement sequences | o | o | o | o |
| Strength training (e.g. Thera-Band, dumbbells, device-supported training) | o | o | o | o |
| Endurance training (e.g. hand bike, ergometer) | o | o | o | o |
| Standing, balance training (also sitting) | o | o | o | o |
| Walking training, treadmill training | o | o | o | o |
| Wheelchair training (e.g. overcoming curbs) | o | o | o | o |
| Transfer training out of and into the wheelchair | o | o | o | o |
| Mobility, everyday life training (e.g. overcoming stairs) | o | o | o | o |
| Own exercises / homework program | o | o | o | o |
| Other (please specify): | o | o | o | o |

| The following statements relate to how you rate your physiotherapy practice, your prescriptions for physiotherapy, and the overall content of your physiotherapy. The prescriptions and treatment contents refer exclusively to the time after your inpatient rehab. Even if you are not currently receiving physiotherapy, please answer the questions in relation to the physiotherapy treatments you have received. | | | | | | | | | | | |
| --- | --- | --- | --- | --- | --- | --- | --- | --- | --- | --- | --- |
|  | | Completely true | | True | | Neutral | | Not true | | Not true at all | Unclear/n.a. |
| Overall, I rate my physiotherapy practice as very good. | | o | | o | | o | | o | | o | o |
| Overall, I rate my previous prescriptions of my physiotherapy as very good. | | o | | o | | o | | o | | o | o |
| Overall, I rate my previous physiotherapy content as very good. | | o | | o | | o | | o | | o | o |
|  | | | | | | | | | | | |
| The following statements refer to your physiotherapist(s). Your own physiotherapist is the one who treats you most often for your SCI. If you are treated equally by two or more physiotherapists, choose one to which you relate your answers. The comparison between different physiotherapists refers to regular treatments by different physiotherapists. Even if you are not currently receiving physiotherapy, please answer the questions related to your recent experience with physiotherapists. | | | | | | | | | | | |
| How many physiotherapists treat you regularly in your physiotherapy practice? | One physiotherapist\|several physiotherapists (How many:…) | | | | | | | | | | |
|  | Completely true | | True | | Neutral | | Not true | | Not true at all | | Unclear/n.a. |
| I know my physiotherapist very well. | o | | o | | o | | o | | o | | o |
| My physiotherapist knows my medical history very well. | o | | o | | o | | o | | o | | o |
| My physiotherapist always remembers what he/she did at my last visit. | o | | o | | o | | o | | o | | o |
| My physiotherapist knows my family circumstances very well. | o | | o | | o | | o | | o | | o |
| My physiotherapist knows my home environment very well | o | | o | | o | | o | | o | | o |
| My physiotherapist knows very well what I do in my daily life. | o | | o | | o | | o | | o | | o |
| My physiotherapist is very aware of what I think is important when it comes to my treatment. | o | | o | | o | | o | | o | | o |
| I will be fully involved in decisions regarding my treatment. | o | | o | | o | | o | | o | | o |
| My physiotherapist has extensive medical expertise and understanding of SCI/stroke. | o | | o | | o | | o | | o | | o |
| My physiotherapist can advise me very well beyond physiotherapy treatment (e.g. everyday questions, provision of aids, sports for the disabled, training for relatives). | o | | o | | o | | o | | o | | o |
| I am very satisfied with my physiotherapist. | o | | o | | o | | o | | o | | o |
| My physiotherapist organizes the therapy sessions very diversified. | o | | o | | o | | o | | o | | o |
| Variations in physiotherapy is exceptionally good for me. | o | | o | | o | | o | | o | | o |
| I benefit a lot from being treated by different physiotherapists. | o | | o | | o | | o | | o | | o |
| The content of my ambulatory physiotherapy is very different from that of my physiotherapy in inpatient rehabilitation. | o | | o | | o | | o | | o | | o |
| During my inpatient rehabilitation, the focus was very much on everyday functions. | o | | o | | o | | o | | o | | o |
| In my ambulatory physiotherapy, the focus is very much on everyday functions. | o | | o | | o | | o | | o | | o |

## Part 4: Collaboration between your doctor and your physiotherapist

| The following statements relate to how your treating physician and your own physiotherapist work together. Your doctor is the one who prescribes physiotherapy. Your physiotherapist is the one who treats you most often for your SCI. | | | | | | |
| --- | --- | --- | --- | --- | --- | --- |
|  | Completely true | True | Neutral | Not true | Not true at all | Unclear/n.a. |
| My doctor and my physiotherapist share information very well. | o | o | o | o | o | o |
| Information between my doctor and physiotherapists is mainly communicated by me. | o | o | o | o | o | o |
| My doctor and my physiotherapist work very well together. | o | o | o | o | o | o |
| There are no discrepancies between my doctor and physiotherapist regarding the further prescription of physiotherapy. | o | o | o | o | o | o |
| The care provided by my doctor and my physiotherapist is very well coordinated. | o | o | o | o | o | o |
| My doctor or physiotherapist always know very well what the other is doing. | o | o | o | o | o | o |
| Close collaboration between my doctor and physiotherapist is very important to my care. | o | o | o | o | o | o |
| I consider the cooperation between my doctor and physiotherapist to be very good. | o | o | o | o | o | o |
|  | | | | | | |
| Part 5: Your overall impression of your health care | | | | | | |
| The following statements relate to your assessment and experience of your current personal health care. | | | | | | |
|  | Completely true | True | Neutral | Not true | Not true at all | Unclear/n.a. |
| In general, I consider the range of health services in my region to be very good. | o | o | o | o | o | o |
| In general, I rate my ambulatory physiotherapy as very good. | o | o | o | o | o | o |
| In general, I rate my ambulatory occupational therapy as very good. | o | o | o | o | o | o |
| In general, I rate my ambulatory speech therapy as very good. | o | o | o | o | o | o |
| In general, I rate the service and advice provided by my medical supply store as very good. | o | o | o | o | o | o |
| In general, I consider my mobility promoting assistive devices (e.g. wheelchair, crutches) to be very good. | o | o | o | o | o | o |
| In general, I rate my equipment of assistive devices aids for everyday life (e.g. toilet seat raiser, gripper) as very good. | o | o | o | o | o | o |
| I am fully involved in decisions regarding my medical treatments. | o | o | o | o | o | o |
| In general, it is very easy to get the necessary physiotherapy on a regular basis. | o | o | o | o | o | o |
| In general, it is very easy to obtain my necessary assistive devices for mobility and/or daily living. | o | o | o | o | o | o |
| In general, it is very easy to consult a doctor / therapist of my choice. | o | o | o | o | o | o |
| In general, it is very easy to obtain reimbursement for therapy, medical aids or other health services. | o | o | o | o | o | o |

| It would have been important to me to get the following measures. Please check all that apply. | Home treatments (home visits)\|Ambulatory therapy (Occupational Therapy\|Voice or speech therapy\|Other (please specify): )\|Assistive devices for mobility (e.g. wheelchair)\|Aids for daily living (washing, dressing, toileting)\|Other aids (please specify): \|Help with remodeling or furnishing my home\|Special equipment for the workplace\|Transition to working life\|Counseling for working life\|Other (please specify): \|I have received everything I need. |
| --- | --- |

# Questionnaire 3: Patient-reported treatment goal attainment (implemented into App)

### Patient goals

| Think about the upcoming prescription period. | | | | | |
| --- | --- | --- | --- | --- | --- |
| The following treatment goals are to be achieved: | | | | | |
|  | Strongly agree | Agree | Undecided | Disagree | Strongly Disagree |
| 1. maintenance/ improvement of joint mobility |  |  |  |  |  |
| 2. pain relief |  |  |  |  |  |
| 3. reduction of spasticity in the legs |  |  |  |  |  |
| 4. maintenance/ improvement of strength and endurance |  |  |  |  |  |
| 5. maintenance/ improvement of positioning and transferring |  |  |  |  |  |
| 6. maintenance/ improvement of standing, balance |  |  |  |  |  |
| 7. maintenance/ improvement of walking |  |  |  |  |  |
| 8. maintenance/ improvement of wheelchair mobility |  |  |  |  |  |
| 9. maintenance/ improvement of mobility in the apartment/ house |  |  |  |  |  |
| 10. maintaining/ improving mobility immediately around the apartment/ house (e.g., garden, sidewalk in front of apartment/ house) |  |  |  |  |  |
| 11. maintenance/ improvement of mobility beyond the dwelling (e.g. to shopping, to the doctor, to work) |  |  |  |  |  |

### Therapist goals

| Think about the upcoming prescription period. | | | | | |
| --- | --- | --- | --- | --- | --- |
| The following treatment goals are to be achieved: | | | | | |
|  | Strongly agree | Agree | Undecided | Disagree | Strongly Disagree |
| 1. maintenance/ improvement of joint mobility |  |  |  |  |  |
| 2. pain relief |  |  |  |  |  |
| 3. reduction of spasticity in the legs |  |  |  |  |  |
| 4. maintenance/ improvement of strength and endurance |  |  |  |  |  |
| 5. maintenance/ improvement of positioning and transferring |  |  |  |  |  |
| 6. maintenance/ improvement of standing, balance |  |  |  |  |  |
| 7. maintenance/ improvement of walking |  |  |  |  |  |
| 8. maintenance/ improvement of wheelchair mobility |  |  |  |  |  |
| 9. maintenance/ improvement of mobility in the apartment/ house |  |  |  |  |  |
| 10. maintaining/ improving mobility immediately around the apartment/ house (e.g., garden, sidewalk in front of apartment/ house) |  |  |  |  |  |
| 11. maintenance/ improvement of mobility beyond the dwelling (e.g. to shopping, to the doctor, to work) |  |  |  |  |  |

### Patient: therapy evaluation

| Think about the past prescription period. | | | | | | | |
| --- | --- | --- | --- | --- | --- | --- | --- |
| You have achieved the following therapy goals: | | | | | | | |
|  | Strongly agree | Agree | | Undecided | Disagree | | Strongly disagree |
| 1. maintenance/ improvement of joint mobility |  |  | |  |  | |  |
| 2. pain relief |  |  | |  |  | |  |
| 3. reduction of spasticity in the legs |  |  | |  |  | |  |
| 4. maintenance/ improvement of strength and endurance |  |  | |  |  | |  |
| 5. maintenance/ improvement of positioning and transferring |  |  | |  |  | |  |
| 6. maintenance/ improvement of standing, balance |  |  | |  |  | |  |
| 7. maintenance/ improvement of walking |  |  | |  |  | |  |
| 8. maintenance/ improvement of wheelchair mobility |  |  | |  |  | |  |
| 9. maintenance/ improvement of mobility in the apartment/ house |  |  | |  |  | |  |
| 10. maintaining/ improving mobility immediately around the apartment/ house (e.g., garden, sidewalk in front of apartment/ house) |  |  | |  |  | |  |
| 11. maintaining/ improving mobility beyond the dwelling (e.g., to shopping, to the doctor, to work) |  |  | |  |  | |  |
| 12. global assessment:  Were you satisfied with the treatment | Yes | | No opinion | | | No | |

### Therapist: therapy evaluation

| Think about the past prescription period. | | | | | |
| --- | --- | --- | --- | --- | --- |
| You have achieved the following therapy goals: | | | | | |
|  | Strongly agree | Agree | Undecided | Disagree | Strongly disagree |
| 1. maintenance/ improvement of joint mobility |  |  |  |  |  |
| 2. pain relief |  |  |  |  |  |
| 3. reduction of spasticity in the legs |  |  |  |  |  |
| 4. maintenance/ improvement of strength and endurance |  |  |  |  |  |
| 5. maintenance/ improvement of positioning and transferring |  |  |  |  |  |
| 6. maintenance/ improvement of standing, balance |  |  |  |  |  |
| 7. maintenance/ improvement of walking |  |  |  |  |  |
| 8. maintenance/ improvement of wheelchair mobility |  |  |  |  |  |
| 9. maintenance/ improvement of mobility in the apartment/ house |  |  |  |  |  |
| 10. maintaining/ improving mobility immediately around the apartment/ house (e.g., garden, sidewalk in front of apartment/ house) |  |  |  |  |  |
| 11. maintenance/ improvement of mobility beyond the dwelling (e.g. to shopping, to the doctor, to work) |  |  |  |  |  |

# Questionnaire 4: therapy measures (implemented into App)

At the end of each prescription period; triggered via evening query.

1) The physical therapist should select the individual therapy measures from the categories ("Controlled environment", "Simulation of everyday life", and "Everyday life") by clicking on them.

2) after that, the previously selected therapy measures should appear evenly distributed in a visual analog scale (0-100%). The physical therapist should determine what the percentage of his therapy was and adjust the individual sliders. (Example see below)

3) technical features

It should be determined if the sliders were adjusted at all. If no slider was adjusted, a safety question should appear: "Did you do all therapy measures equally?" Yes = completion questionnaire completed, No = sliders need to be moved.

|  | Therapy measures |
| --- | --- |
| Controlled environment  in physiotherapy practice rooms | Passive movement of joints |
|  | Endurance training |
|  | Strength training |
|  | Mobility on treatment table, sitting/sitting balance |
|  | Standing/balance training |
|  | Gait training |
|  | Transfer training and/or wheelchair training |
| Simulation of everyday life (in practice rooms)  e.g., walking over changing surfaces (carpet/slippery floor), transfer from floor to wheelchair, handling of wheelchair | Climbing stairs, gait training |
|  | Transfer training and/or wheelchair training |
| Everyday life  e.g. transfer to car or home toilet, walking in home environment or on sidewalk, boarding public transportation | Climbing stairs, gait training |
|  | Transfer training and/or wheelchair training |

Example:

The physiotherapist selects the therapy measure "Endurance training" from the category "Controlled environment", the therapy measure "Stair climbing, walking" from the category " Simulation everyday life" and the therapy measure "wheelchair training" from the category " Everyday life". The selected therapy measures then appear in the visual analog scale as follows:

| Endurance training | Climbing Stairs, Walking | Wheelchair training |
| --- | --- | --- |
| 33.3% | 33.3% | 33.3% |

0% **↑** **↑** 100% Slider Slider

Now the physiotherapist should adjust the controllers according to his/her percentage in the therapy.

| Endurance training | Climbing Stairs, Walking | Wheelchair training |
| --- | --- | --- |
| 50% | 30% | 20% |

0% **↑** **↑** 100% Slider Slider
